# Temporo-spatial variations in resistance determinants and clonality of *Acinetobacter baumannii* and *Pseudomonas aeruginosa* strains from Romanian hospitals and wastewaters

**DOI:** 10.1186/s13756-022-01156-1

**Published:** 2022-09-14

**Authors:** Irina Gheorghe-Barbu, Ilda Czobor Barbu, Laura Ioana Popa, Grațiela Grădișteanu Pîrcălăbioru, Marcela Popa, Luminița Măruțescu, Mihai Niță-Lazar, Alina Banciu, Cătălina Stoica, Ștefania Gheorghe, Irina Lucaciu, Oana Săndulescu, Simona Paraschiv, Marius Surleac, Daniela Talapan, Andrei Alexandru Muntean, Mădălina Preda, Mădălina-Maria Muntean, Cristiana Cerasella Dragomirescu, Mircea Ioan Popa, Dan Oțelea, Mariana Carmen Chifiriuc

**Affiliations:** 1 Department of Microbiology and Immunology, Faculty of Biology, University of Bucharest, Bucharest, Romania; 2 Research Institute of the University of Bucharest (ICUB), Bucharest, Romania; 3 National Institute of Research and Development for Biological Sciences, Bucharest, Romania; 4 National Research and Development Institute for Industrial Ecology (INCD ECOIND), Bucharest, Romania; 5 National Institute for Infectious Diseases ‘Matei Bals’, Bucharest, Romania; 6 University of Medicine and Pharmacy “Carol Davila”, Bucharest, Romania; 7 “Cantacuzino” National Medical-Military Research and Development Institute, Bucharest, Romania; 8 Romanian Academy, 050045 Bucharest, Romania

**Keywords:** Antimicrobial resistance, Nonfermenting gram-negative Bacilli, Nosocomial infections, Wastewater, Epidemic clones

## Abstract

**Background:**

Romania is one of the European countries reporting very high antimicrobial resistance (AMR) rates and consumption of antimicrobials. We aimed to characterize the AMR profiles and clonality of 304 multi-drug resistant (MDR) *Acinetobacter baumannii* (*Ab*) and *Pseudomonas aeruginosa* (*Pa*) strains isolated during two consecutive years (2018 and 2019) from hospital settings, hospital collecting sewage tanks and the receiving wastewater treatment plants (WWTPs) located in the main geographical regions of Romania.

**Methods:**

The strains were isolated on chromogenic media and identified by MALDI-TOF-MS. Antibiotic susceptibility testing and confirmation of ESBL- and CP- producing phenotypes and genotypes were performed. The genetic characterization also included horizontal gene transfer experiments, whole-genome sequencing (WGS), assembling, annotation and characterization.

**Results:**

Both clinical and aquatic isolates exhibited high MDR rates, especially the *Ab* strains isolated from nosocomial infections and hospital effluents. The phenotypic resistance profiles and MDR rates have largely varied by sampling point and geographic location. The highest MDR rates in the aquatic isolates were recorded in Galați WWTP, followed by Bucharest. The *Ab* strains harbored mostly *bla*_OXA-23_, *bla*_OXA-24_, *bla*_SHV_, *bla*_TEM_ and *bla*_GES_, while *Pa* strains *bla*_IMP_, *bla*_VIM_, *bla*_NDM_, *bla*_VEB_, *bla*_GES_ and *bla*_TEM_, with high variations depending on the geographical zone and the sampling point. The WGS analysis revealed the presence of antibiotic resistance genes (ARGs) to other antibiotic classes, such as aminoglycosides, tetracyclines, sulphonamides, fosfomycin, phenicols, trimethoprim-sulfamethoxazole as well as class 1 integrons. The molecular analyses highlighted: (i) The presence of epidemic clones such as ST2 for *Ab* and ST233 and ST357 for *Pa*; (ii) The relatedness between clinical and hospital wastewater strains and (iii) The possible dissemination of clinical *Ab* belonging to ST2 (also proved in the conjugation assays for *bla*_OXA-23_ or *bla*_OXA-72_ genes), ST79 and ST492 and of *Pa* strains belonging to ST357, ST640 and ST621 in the wastewaters.

**Conclusion:**

Our study reveals the presence of CP-producing *Ab* and *Pa* in all sampling points and the clonal dissemination of clinical *Ab* ST2 strains in the wastewaters. The prevalent clones were correlated with the presence of class 1 integrons, suggesting that these isolates could be a significant reservoir of ARGs, being able to persist in the environment.

**Supplementary Information:**

The online version contains supplementary material available at 10.1186/s13756-022-01156-1.

## Background

Antimicrobial resistance (AMR) is an increasing worldwide concern. Romania is one of the European countries reporting elevated AMR rates and the country with the third highest consumption of antibacterials for systemic use in the community sector (according to data from 2020) [1]. Antibiotics are one of the most popular pharmaceuticals used in human medicine, veterinary care, and farming [2–4]. Unfortunately, antibiotics are also frequent contaminants in domestic wastewater, municipal sewage and wastewater treatment plant (WWTP) effluents from where they dissipate into the environment [5–7]. Hospitals generate huge amounts of wastewater daily, high in pathogenic microorganisms, antibiotics and other pharmaceutical or toxic substances, discharged in urban wastewater systems. Hospital wastewaters, coupled with urban, industrial etc. wastewaters reach the WWTPs turning them in key sources of both antibiotic resistant bacteria (ARB) and antibiotic resistant genes (ARGs). ARGs are disseminated via mobile genetic elements (MGEs) to other non-resistant bacterial strains [8–10] during wastewater treatment. The WWTPs standard procedures only partially remove ARGs, ARBs and other resilient pollutants. The remaining contaminants are contributing to the pollution of the natural environments, facilitating the selection and dissemination of ARGs and ARB [7, 11–13] into crops, animals, and humans, from which they could be (re)introduced into the medical environment [14]. Also, the ARGs can be transmitted to the aquatic microbiota via horizontal gene transfer (HGT), being increased in natural environment bacterial biofilms and under pharmaceutical and heavy metal contamination induced stress [15].

The lack of surveillance of non-clinical reservoirs is considered one of the main contributors to the spread of AMR, particularly in developing countries. In this context, during the last few years, international authorities have made considerable efforts to improve the monitoring of AMR in different environments, underlining the necessity to strengthen intersectoral human, animal, and agricultural cooperation. One of the priority topics of The Joint Programme Initiative on Antimicrobial Resistance (JPIAMR) is represented by the elucidation of the role of the environment as a source for the selection and dissemination of AMR. One important goal is mapping the distribution of multidrug-resistant (MDR) pathogens and plasmids of different genomic lineages in different clinical and aquatic compartments. This important insight could be translated into policy measures to monitor AMR and control the emergence and spread of ARB [16, 17].

*Acinetobacter baumannii* (*Ab*) and *Pseudomonas aeruginosa* (*Pa*) are members of the initially designed “ESKAPE”, then “ESCAPE” (*Enterococcus faecium*, *Staphylococcus aureus*; *Clostridioides difficile*, *Acinetobacter baumannii*, *Pseudomonas aeruginosa*, and *Enterobacteriaceae*) group [18, 19]. Centers for Disease Control and Prevention (CDC) classified them urgent threat due to their often MDR features [20] and therefore requiring concerted research and management efforts [21]. *Ab* and *Pa* exhibit all known AMR mechanisms, such as drug inactivation/alteration, modification of drug binding sites/targets, cell permeability modification and biofilm development [22]. One of the most clinically relevant mechanisms of resistance in *Ab* and *Pa* strains is represented by the production of antibiotic inactivating hydrolytic enzymes, especially carbapenemases (CPs).

So far, five main groups of acquired chromosomal or plasmid located class D β-lactamases (CHDLs) with different geographic distribution have been identified in *Ab* strains, i.e., OXA-23, OXA-24/-40, OXA-58, OXA-143 and OXA-235 [23]. In *Pa* strains, there were identified eleven families of class B metallo-β-lactamases (MBL) [Verona integron-encoded MBL (VIM); imipenemases (IMPs); New Delhi MBL (NDM); Australian imipenemase (AIM); Central Alberta MBL (CAM); Dutch imipenemase (DIM); Florence imipenemase (FIM); German imipenemase (GIM); Hamburg MBL (HMB); São Paulo MBL (SPM) and Seoul imipenemase (SIM)], chromosomal/plasmid encoded or integron-borne, the VIM, IMP and NDM types being distributed worldwide [24].

Presently, there is insufficient data on the *Ab* and *Pa* dissemination and survival from hospitals in wastewater and finally into the natural recipients. Despite the huge burden of AMR presence in Romania and its significant overall impact on European AMR rates, the genetic relationships between the bacterial strains isolated from different aquatic environmental and clinical compartments were not investigated at a national level.

We aimed to perform a phenotypic and molecular characterization of the acquired resistome of a significant number of MDR *Ab* and *Pa* strains isolated in the same temporal sequence from the hospital environments and the receiving wastewater network from different counties in Romania.

## Methods

### Phenotypic characterization of the *Ab* and *Pa* strains

#### Sampling location

**Fig. 1:**
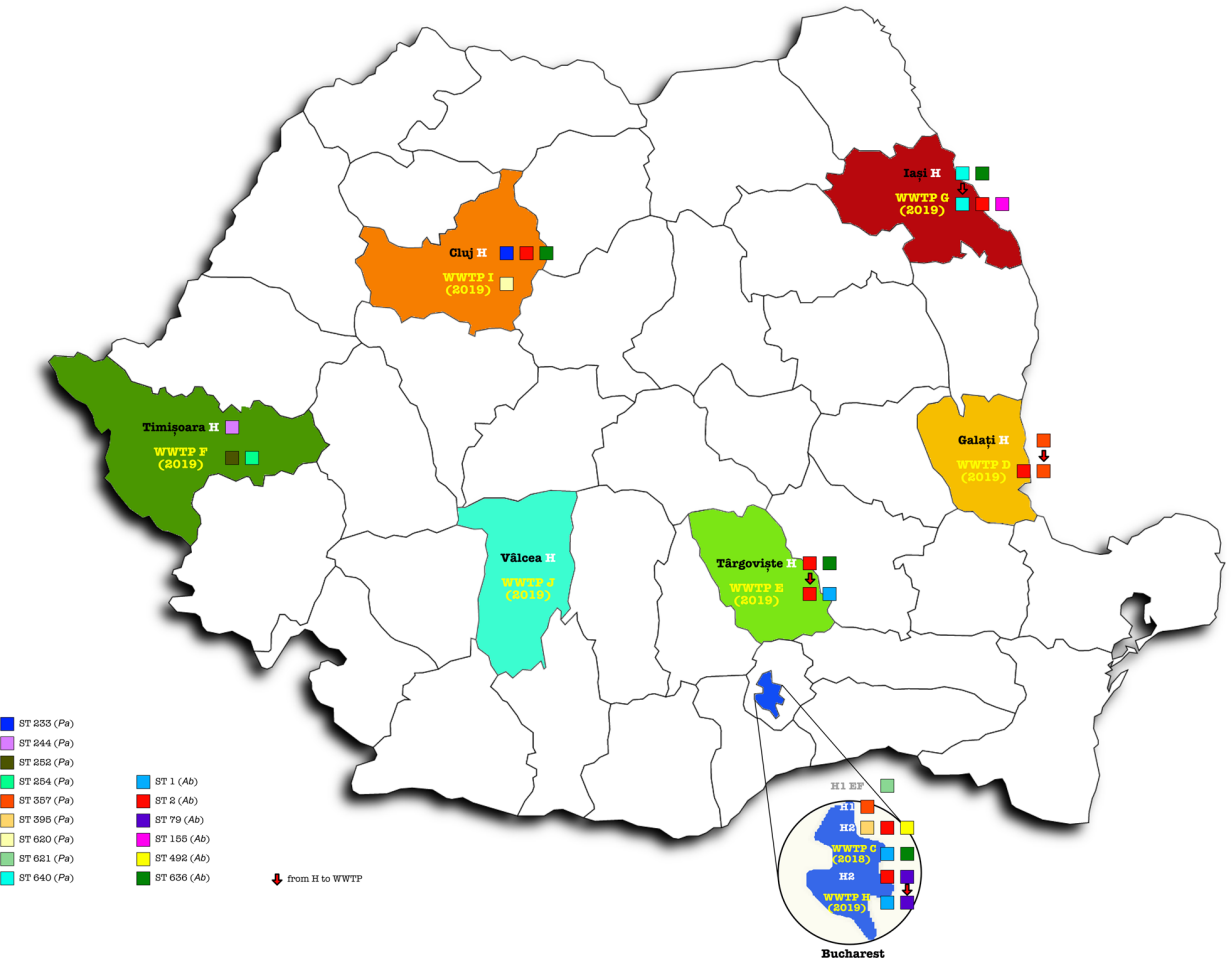
Geographic distribution of the sampling points

Water sampling was performed from September 2018 to August 2019. The collection points were represented by sewage tanks from eight hospital units and their wastewater collecting WWTPs. The eight sampling locations covered several Romanian regions such as the Southern region, with sampling locations in Bucharest (C/H—Glina municipal WWTP 2018/2019), WWTP Târgoviște (E) and WWTP Râmnicu-Vâlcea (J); Central and Western regions: WWTP Cluj (I) and WWTP Timișoara (F); Northern and Eastern regions: WWTP Iași (G) and WWTP Galați (D) (Fig. 1).

The different sampling points from the selected locations were: hospital/WWTP effluent (EF), WWTPs influent (IN), activated sludge (AS) and returned sludge (RS), where all isolated strains were considered within a single group.

### Isolation and characterization of *Ab* and *Pa* strains

The water samples were collected in sterile glass containers, transported to the laboratory at 5 ± 3 °C and processed within the first 24 h. The water samples were diluted and filtered through 0.45 μm pore size membrane filters (Millipore, France), as described in SR EN ISO 9308–2/2014 (for coliform bacteria) and then cultivated on ChromID ESBL agar and ChromID CARBA agar (BioMérieux, France). The resistant colonies developed after cultivation at 37 °C for 24 h in aerobic conditions were subsequently inoculated on the corresponding antibiotic-enriched media for the confirmation of ESBL-(ChromID ESBL) or CP- (ChromID CARBA) producing phenotypes. All resistant strains were identified by MALDI-TOF-MS (Bruker system). In the same time frame with the collection of the water samples (i.e., during a ten-day period prior to the water sampling), *Ab* and *Pa* clinical strains were isolated from intra-hospital infections that occurred in the units discharging the wastewater in the sampled WWTPs.

The antibiotic susceptibility profiles of the identified strains (220 *Ab* and 84 Pa), were determined using the standard disc diffusion method according to Clinical and Laboratory Standards Institute (CLSI guidelines) for 2018 and 2019 [25, 26] (see Additional file 1: Tables S1 and S2).

### PCR for ESBL and CP genes

The strains were *screened* for CP (*bla*_VIM_, *bla*_IMP_, *bla*_NDM_, *bla*_KPC_ for *Pa*), and *bla*_OXA-51/69-like_, *bla*_OXA-23-like_, *bla*_OXA-24-like_, *bla*_OXA-58-like_, *bla*_OXA-143_, *bla*_OXA-235,_ *bla*_NDM_ genes in the case of *Ab* strains) and ESBL encoding genes (*bla*_CTX-M_, *bla*_TEM_, *bla*_SHV_, *bla*_PER_, *bla*_VEB_, *bla*_GES_ for strains belonging to both species) using previously described primers and PCR protocols [27, 28].

### Mating experiments

Transferability of *bla*_OXA-23_ and *bla*_OXA-24_ genes by conjugation was tested using the solid mating method, with rifampicin (RIF) resistant *Acinetobacter baylyi* ADP1 as recipient. Briefly, equal amounts (100 µL) of overnight cultures of the donors (n = 40 *Ab* strains from all isolation sampling points) and recipient strains were mixed and incubated in Brain heart infusion agar plates. Cells were resuspended in saline solution and selected in plates containing RIF (300 mg/L) and meropenem (MEM) (0.5 mg/L) [29]. Characterization of the transconjugants was conducted by PCR.

### Whole-genome sequencing (WGS), assembling, annotation and characterization

To determine the genetic relationships between the clinical and wastewater isolates, 54 strains (*Ab*, n = 34 and *Pa,* n = 20) were selected for WGS based on the isolation source, geographical region, temporal sequence and the presence of MDR phenotype in order to have a complete picture of the antibiotic resistance in different Romanian regions. Total DNA was isolated using DNeasy UltraClean Microbial Kit (Qiagen) and subjected to Illumina (Nextera DNA Flex Library Prep Kit) sequencing on a MiSeq platform (V3, 600 cycles). The sequencing quality was very good (with an average of 88% over Q30 and 95% over Q20 for *Ab* (78% over Q30 and 89% over Q20 for *Pa*), and an average of 1.53 million reads per sample for *Ab* and 1.66 million reads per sample for *Pa*. Raw reads were checked for quality using FastQC v0.18.8 [30], assembled using Shovill v1.1.0 pipeline [31] and primarily annotated using Prokka v.1.14.6 [32], while the prediction of resistance profiles was performed by using ABRicate v0.5 [33], ResFinder, PlasmidFinder v2.1.1 [34–37], PathogenFinder [38], CARD [39]. Strain relatedness was investigated using MultiLocus Sequence Typing (MLST v2.9) [35, 40] and Single Nucleotide Polymorphism (SNP) (v3.1) [41]. Comparative gene analyses were performed using Roary v.3.13.0 [42] and the output was used to infer phylogenies using RAxML v8.2.12 (Maximum Likelihood inference using bootstrap value N = 1000) [43] and visualized using iTOL [44]. The assembled sequences have been deposited in GenBank with BioProject ID PRJNA841266.

The 34 selected *Ab* WGS assemblies were subjected to phylogenetic analysis to further attain their relationship to other *Ab* strains from the NCBI database. For this, all *Ab* sequences available (n = 4175) were downloaded from NCBI and from those, 71 were randomly selected. MLST predictions and annotations were performed on this dataset and, together with the 34 selected strains, were subjected to further pangenome analysis using Roary and aligned with Mafft v.7.741. The resulted phylogenetic tree was drawn with FigTree v.1.4.4 and the final form of the supplementary image was then represented using Affinity Designer v.1.10.5.

## Results

### Identification and antibiotic susceptibility profiles of *Ab* and *Pa* strains

A total number of 220 *Ab* and 84 *Pa* were isolated from clinical and water samples during the two consecutive years.

Two pilot sampling campaigns were performed in Bucharest in 2018 from which 66 *Ab* and 50 *Pa* strains were isolated (Table 1).

**Table 1:**
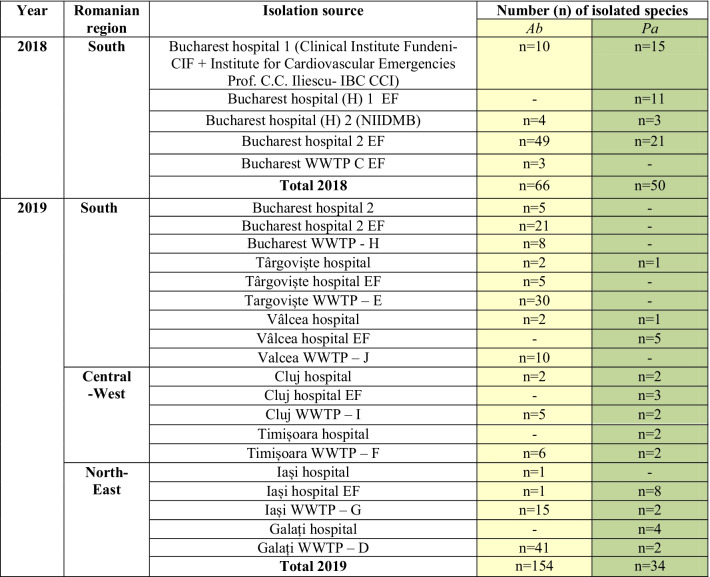
The distribution of *Ab* and *Pa* strains selected for this study

In 2019, the sampling campaign was extended, including, in addition to Bucharest, six other cities that are representative of the main country regions, i.e., North-East (Iași, Galați), Central-West (Cluj, Timișoara) and South (Târgoviște, Râmnicu Vâlcea). A total of 154 *Ab* and 34 *Pa* resistant isolates were recovered (Table 1).

Within the same time frame, clinical *Ab* and *Pa* clinical strains were isolated in hospital units from which wastewater samples were collected. The hospital wastewater was collected and treated by the corresponding WWTPs from the same town.

The analysis of MDR rates from the hospital to the collecting WWTP in the first sampling campaign in 2018 has revealed the following aspects: (1) *clinical isolates*—all *Ab* and the majority of *Pa* strains (93.3–100%) were MDR (Additional file 1: Tables S1 and S2); (2) *hospital EF*—all *Ab* isolates were MDR (Additional file 1: Table S1), while the *Pa* strains exhibited various MDR rates (from 25 to 100%) (Additional file 1: Table S2); (3) *WWTP C, collecting the two hospital EFs—*all *Ab* isolates were MDR (Additional file 1: Table S1).

In 2019, the MDR rates from hospital to the collecting WWTP were as follows: (1) *clinical isolates—*with one exception, all *Ab* isolates were MDR (Additional file 1: Table S1), while the *Pa* strains expressed a high variation of MDR rate (from 0 to 100%) within and between the geographical locations (Additional file 1: Table S2); (2) *hospital EF* – all *Ab* isolates were MDR (Additional file 1: Table S1), while the *Pa* strains exhibited various MDR rates (from 0 to 100%) (Additional file 1: Table S2); (3) *WWTPs*, collecting the sewages of the sampled hospital units effluents—the MDR resistance rates varied from 0 to 100% for both *Ab* and *Pa* strains.

### CP and ESBL encoding genes in clinical and environmental *Ab* and *Pa* strains

#### Profiles of CP and ESBL genes from the clinical to the aquatic environment in 2018 *versus* 2019

The *Ab* strains from the clinical settings to the WWTP effluent and receiving river exhibited different profiles of CP and ESBLs in the two consecutive years, i.e.: (1) *clinical strains—Ab* strains recovered in 2018 were OXA-23 and OXA-24 producers (64.28/42.85%) and only 7.14% were positive for *bla*_TEM_, while in 2019, 41.66% of all intra-hospital *Ab* strains were OXA-23 and OXA-24 producers, 25% were positive for *bla*_SHV_, 16.66% for *bla*_VIM_ and *bla*_VEB_ and 8.33% for *bla*_TEM_ and *bla*_GES_ (Additional file 1: Table S1); (2) *hospital sewage—*the identified carbapenem-hydrolyzing class D β-lactamases (CHLDs) were represented in the two consecutive years by OXA-23 (67.34/96.29%) and OXA-24 (2.04/0%), MBLs by VIM (4.08/14.81%) and ESBLs by TEM (20.41/0%), SHV and PER (each one 0/3.70%), GES (0/18.51%) (Additional file 1: Table S1); (3) *WWTPs—*some of the enzymes were common for the strains isolated in 2018 and 2019 [i.e. OXA-24 (57.14/10.25%); OXA-23 (42.85/53.84%); TEM (28.57/10.89%); SHV (14.28/0.64%)], while some other were different [i.e. NDM in *Ab* strains from 2018 (14.28%) and GES (19.87%); VEB (8.97%); CTX-M (2.56%) and PER (0.64%) in 2019].

The CP and ESBL genes identified in the *Pa* strains isolated among the transmission chain from the clinical sector to the WWTP effluent and receiving river in the two consecutive years were the following: (1) *clinical strains*—the *Pa* strains were positive for *bla*_IMP_ (66.66/0%), *bla*_VIM_ (33.33/20%) and *bla*_VEB_ (38.88/30%); (2) *hospital sewage*—in the two consecutive years the following MBLs were identified in the *Pa* strains: VIM (9.37/18.75%); IMP (9.37/0%); NDM (0/12.5%); while the ESBLs were represented by GES (6.25/6.25%); VEB (43.75/12.5%) and TEM (0/31.25%); (3) *WWTPs*—only the *Pa* strains isolated in 2019 were positive for ESBL encoding genes [*bla*_TEM_ (16.66%); *bla*_GES_ and *bla*_VEB_ (8.33% each)] (Additional file 1: Table S2).

#### Geographic distribution of CP and ESBL genes in clinical and water *Ab* and *Pa* isolates

The 2018 pilot study was limited to the Bucharest region, and then it was extended during 2019 to other regions of the country, allowing us to perform a comparative analysis regarding the geographic distribution of the CP and ESBL encoding genes in the *Ab* and *Pa* strains.

Regarding the *Ab* strains, the isolates from the Southern region expressed the broadest spectrum of CP and ESBL encoding genes, both in *clinical* [i.e., *bla*_OXA-23_ (44.44%); *bla*_OXA-24_ (33.33); *bla*_VIM_ (22.22%); *bla*_TEM_ and *bla*_SHV_ (11.11%)] and the *aquatic isolates* [*hospital sewage*: *bla*_OXA-23_ (100%); *bla*_GES_ (19.23%); *bla*_VIM_ (15.38%); *bla*_VEB_ and *bla*_SHV_ (3.84%) and *WWTPs*: i.e. *bla*_OXA-23_ (44.44%); *bla*_GES_ (32.09%); *bla*_TEM_ (18.51%); *bla*_OXA-24_ (14.81%); *bla*_VEB_ (13.58%); *bla*_CTX-M_ (4.93%) and *bla*_SHV_ (1.23%)].

The *Ab* isolates from the Central-Western region revealed the presence of the following CP and ESBL encoding genes: (1) *in clinical settings*, all *Ab* strains were *bla*_VEB_ positive; 50% were *bla*_OXA-23_ and *bla*_OXA-24_ positive; (2) *the aquatic isolates* recovered from the two sampled WWTPs were *bla*_OXA-23_ and *bla*_VEB_ positive (23.07% each of them).

**Fig. 2:**
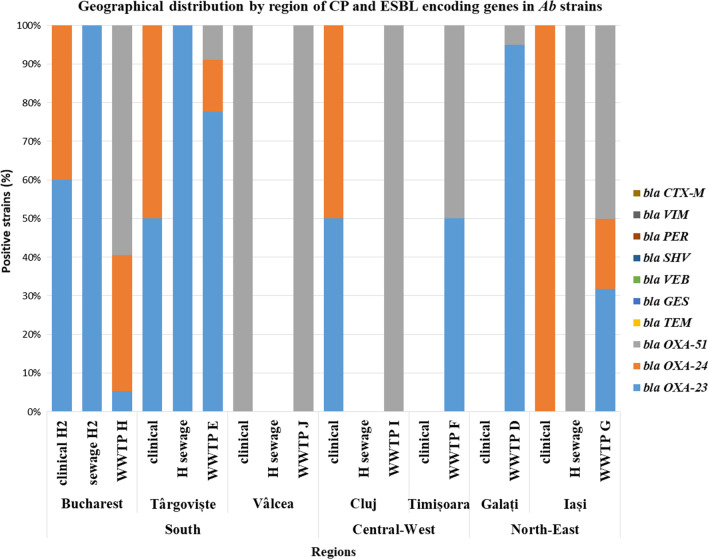
CP and ESBL encoding genes in clinical and wastewater *Ab* from different geographical regions

In the North-Eastern region, the CP and ESBL identified in *Ab* strains from the hospitals to the WWTP were the following: (1) all *clinical Ab* strains were OXA-24 producers; while in the sampled WWTPs, the *Ab* strains harbored OXA-23 (72.58%); OXA-24 (6.45%); TEM (3.22%) and PER (1.61%) (Fig. 2).

**Fig. 3:**
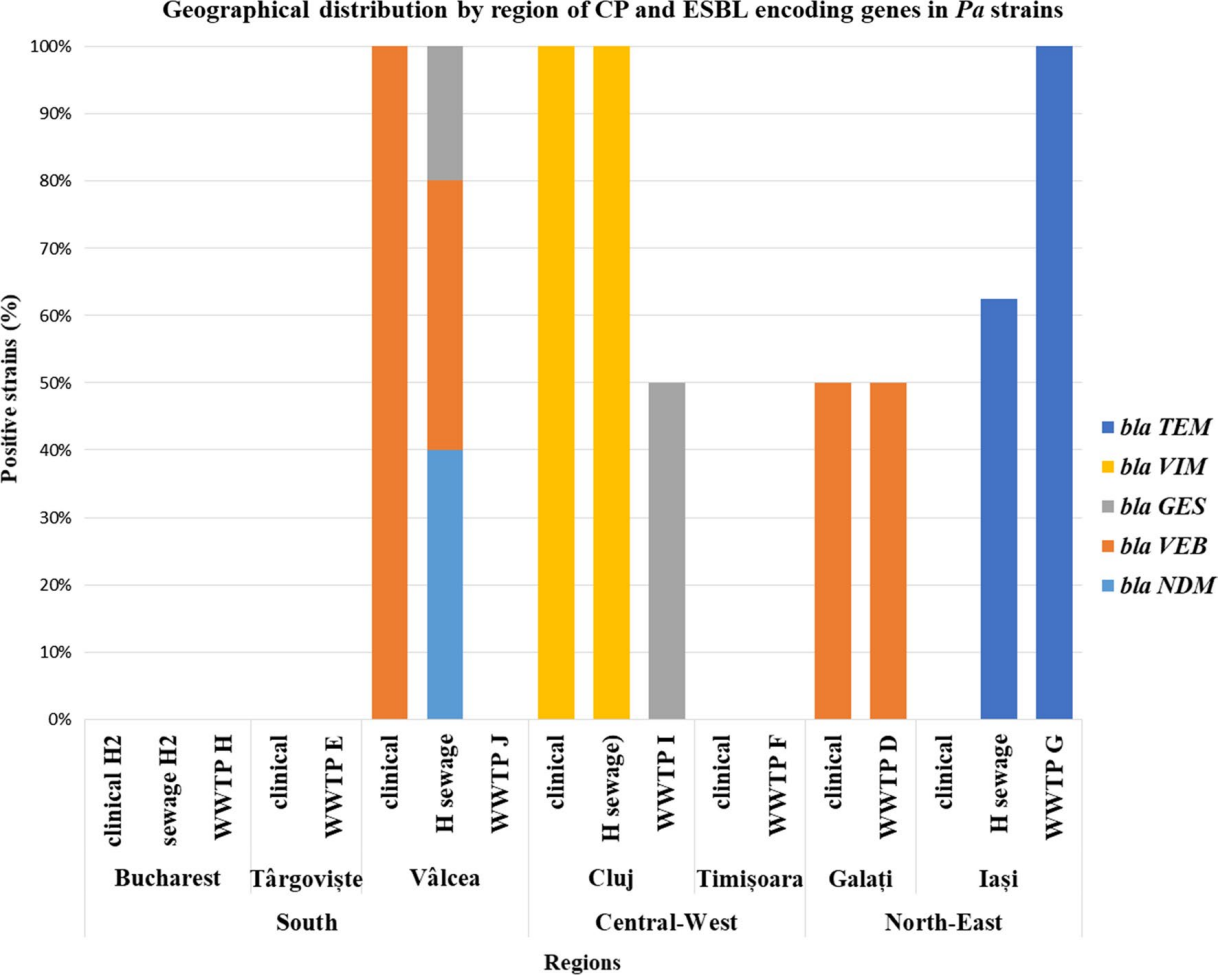
CP and ESBL encoding genes in clinical and wastewater *Pa* from different geographical regions

The geographical distribution of the CP/ESBLs found in *Pa* strains isolated from intra-hospital infections, the hospital sewage tank and the sampled WWTP from the corresponding cities was as follows: in the Southern region, 50% of nosocomial *Pa* strains were VEB producers, while the wastewater *Pa* strains harbored *bla*_VEB_ (40%), _*bla*NDM_ (40%) and *bla*_GES_ (20%); in the North-Eastern region, 50% of clinical *Pa* strains were VEB producers; 62.5% of the hospital sewages strains were positive for *bla*_TEM_; 50% respectively 25% of the WWTPs were *bla*_VEB_ and *bla*_TEM_ positive. The *Pa* strains from the Central-Western regions revealed different CP/ESBLs in clinical/hospital sewage (VIM) and in the sampled WWTPs (14.28% were GES positive) (Fig. 3).

#### WGS analysis of clinical and wastewater *Ab* and *Pa* isolates

In case of nine *Ab* strains recovered in 2018 in Bucharest from intra-hospital infections (n = 2), hospital sewage EF (n = 5) and, the corresponding WWTP EF (n = 2) the WGS demonstrated the presence of OXA-72 and OXA-23 encoding genes in the IN and the EF of the collecting sewage tank and of genes encoding aminoglycoside modifying enzymes (AMEs) i.e. aph(3′)-VIa, ant(3″)-IIa, sulphonamides (sul1) and class 1 integrons (qacE∆1 integron-associated gene in 3′ CS region), in all investigated samples (Table 2).

**Table 2:**
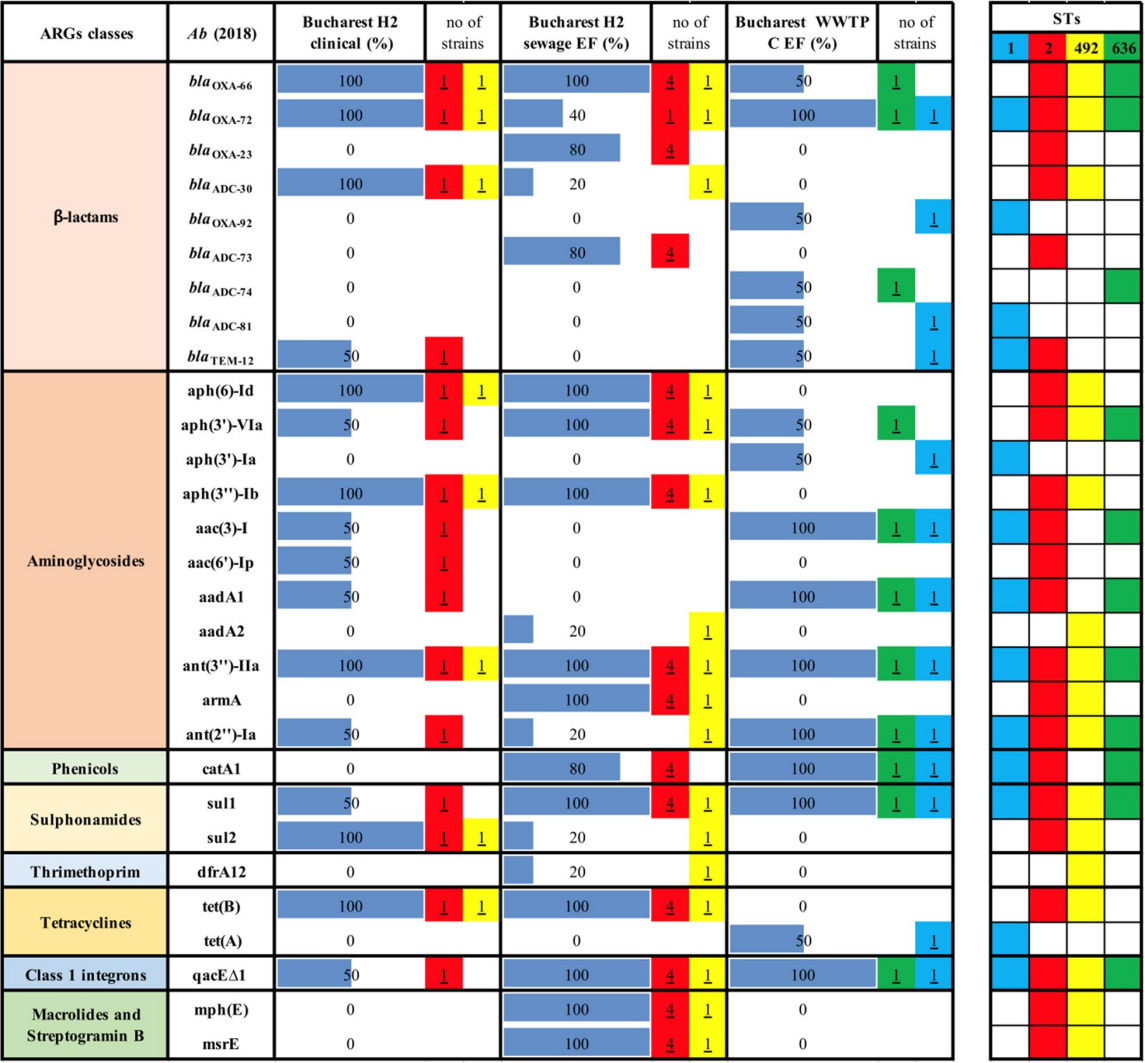
ARGs in clinical and wastewater *Ab* strains isolated in Bucharest in 2018

The WGS analysis of 12 *Ab* clinical strains isolated in 2019 from two hospital units (Bucharest H2 and Târgoviște), the collecting sewage tank of Bucharest hospital 2, and the sampled corresponding WWTPs from Bucharest (H) and Târgoviște (E) revealed the presence of *bla*_OXA-23_ in all sampled points from Bucharest. The presence of both *bla*_OXA-23_ and *bla*_OXA-72_ was shown in clinical *Ab* strains from Târgoviște hospital unit and in the corresponding WWTP (i.e., *bla*_OXA-23_ in EF and *bla*_OXA-72_ in the RS) (Additional file 2: Table S3). Similarly, the AMEs (i.e., aph(6)-Id, ant(3″)-IIa), sulphonamides resistance (sul 1) and class 1 integrons (qacE∆1 integron associated gene) were present in all sampled sites from Bucharest. The WGS of nine *Ab* clinical and wastewater strains from Eastern and Northern Romania revealed a high diversity of ARGs, with differences between different cities, i.e., *bla*_OXA-23_, aph(6)-Id, aph(3″)-Ib, armA, sul 1, tet(B), mph(E), msrE and qacE∆ in Galați WWTP (D) and *bla*_OXA-72,_ aph(3′)-Ia, aac(3)-I, aadA1, ant(2″)-Ia, sul1 and qacE∆1 in Iași WWTP (G) (Additional file 2: Table S3).

The WGS analysis of the acquired resistome of the 10 *Pa* strains recovered in 2018 revealed the dissemination of CP *bla*_IMP-13_ and genes encoding AMEs (aph(3′)-IIb, ant(2″)-Ia), fosfomycin (fosA), phenicols (catB7, bcr1) and trimethoprim-sulfamethoxazole (sul1) resistance genes in strains isolated from one Bucharest hospital and its effluent (Table 3). In the case of the second investigated hospital and the corresponding sewage tank, there has been noticed the presence of genes encoding AMEs (aph(3′)-IIb) and phenicols (catB) (Table 3).

**Table 3:**
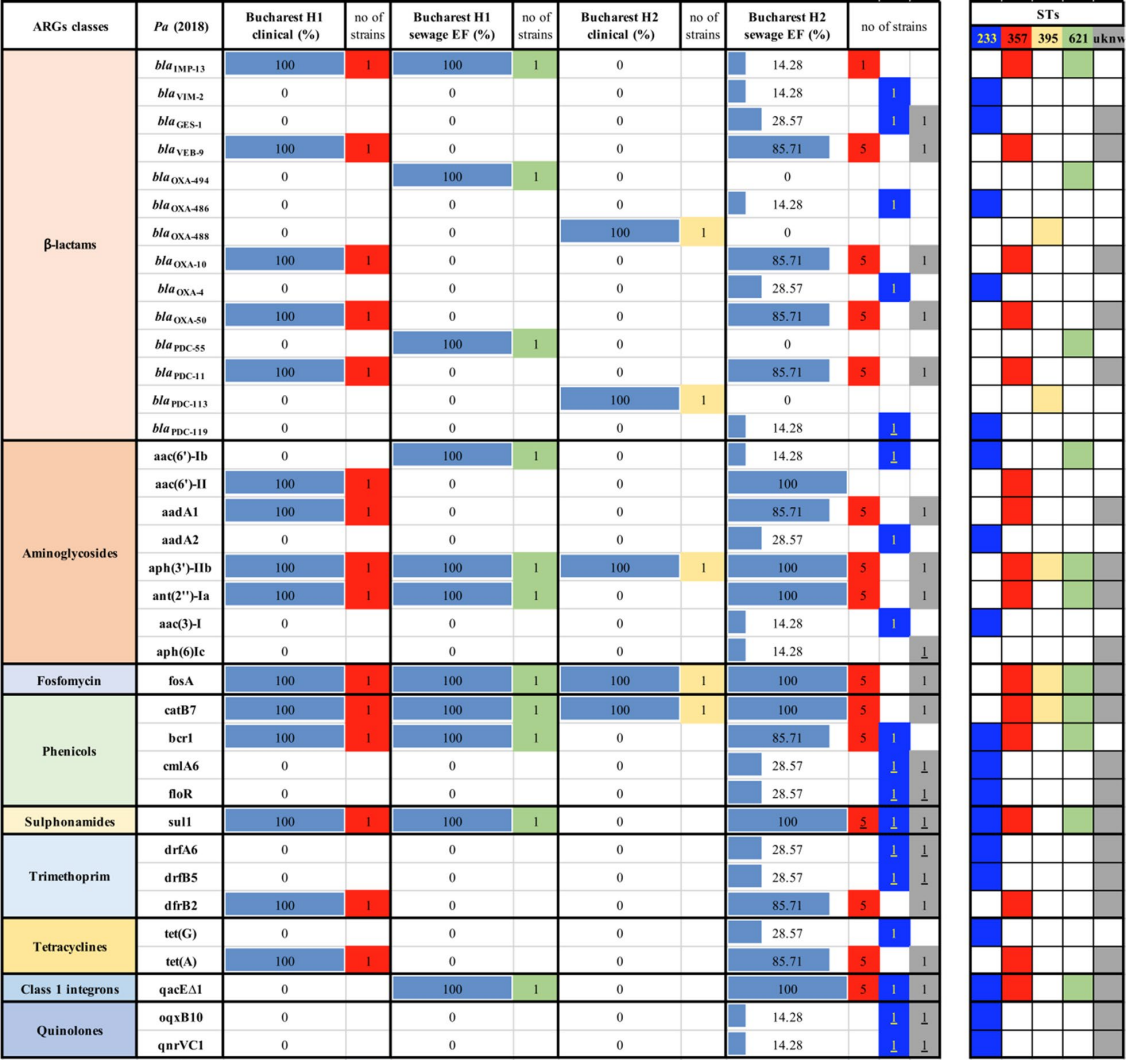
ARGs in clinical and wastewater *Pa* strains isolated in Bucharest in 2018

The ARGs distribution of four *Pa* strains, collected from Northern and Eastern Romania in 2019, revealed the presence of ESBL encoding genes (*bla*_TEM-40_, *bla*_VEB-9_), AMEs encoding genes (aac(6′)-II, aadA1, aph(3′)-IIb) as well as determinants of resistance to fosfomycin (fosA), phenicols (catB7, bcr1), tetracycline [tet(A)] and class 1 integrons (qacE∆1 integron associated gene) in clinical and wastewater samples. Regarding the six *Pa* clinical and wastewater strains from Central and Western regions of Romania, the presence of resistance genes encoding for fosfomycin (fosA) and phenicols (catB7, bcr1) has been observed in *Pa* strains from almost all sources (Table 4).

**Table 4:**
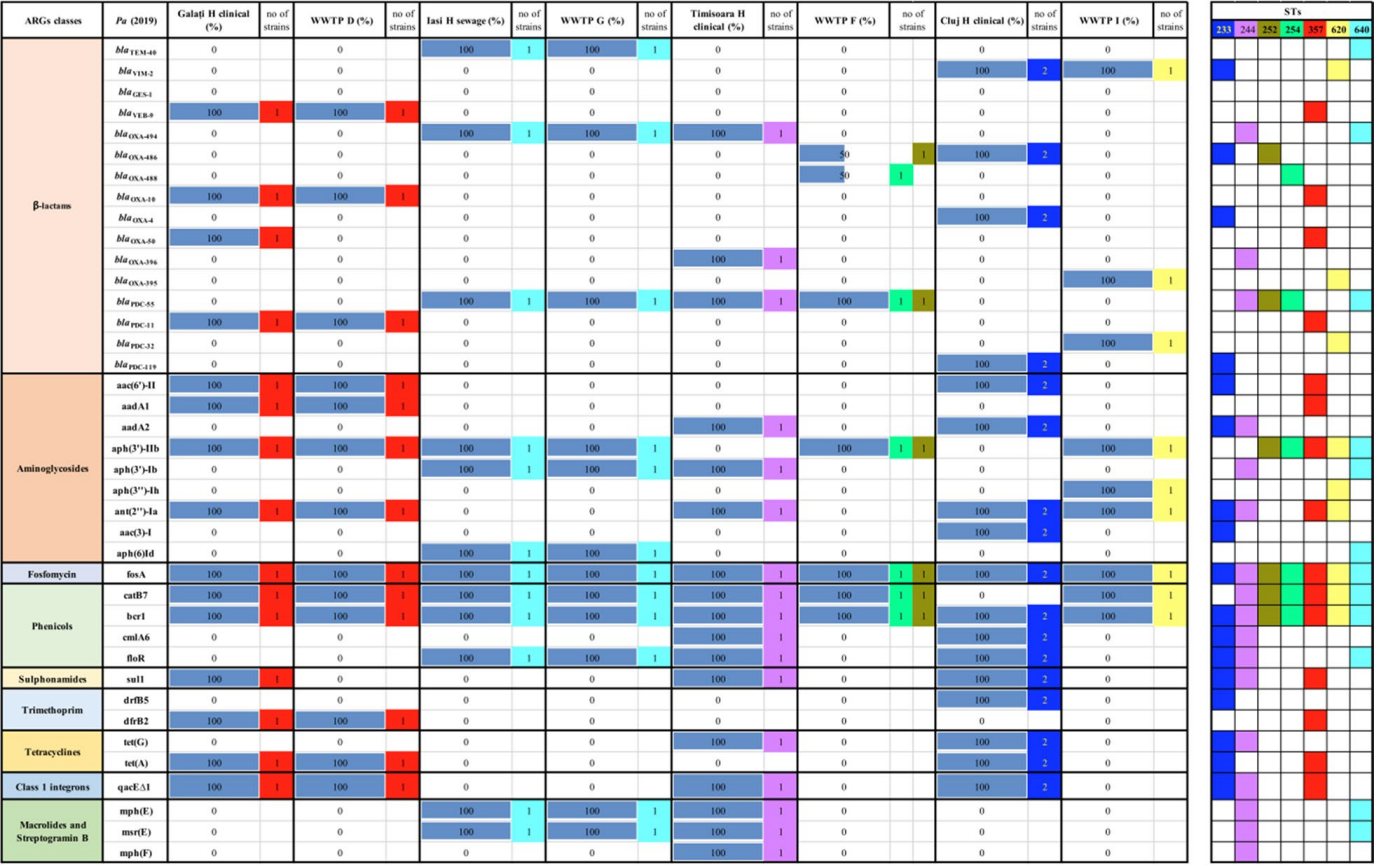
ARGs in clinical and wastewater *Pa* strains isolated from North-Eastern and Central-Western Romania in 2019

### *Ab* and *Pa* strains molecular phylogeny

Based on SNP analyses and MLST profiles, the *Ab* strains were divided in six groups.

**Fig. 4:**
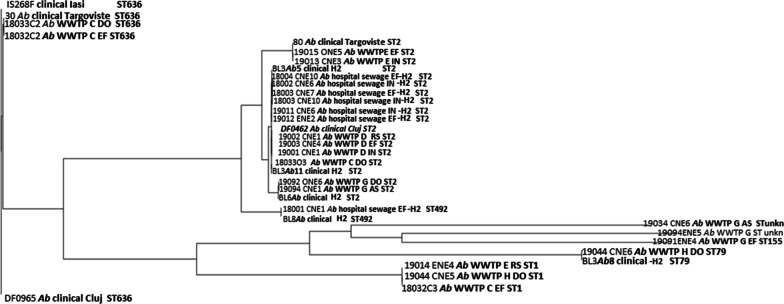
Phylogenetic tree of clinical and wastewater *Ab* strains

Group I included clinical isolates from Iași, Târgoviște and Cluj and the effluent and downstream of Bucharest WWTP C, belonging to ST636. The SNP analysis suggests the similarity between a clinical isolate sampled in 2019 and two aquatic strains from this group, sampled in 2018 (harboring 51 and, respectively, 60 SNPs); group II included most of the strains (clinical and wastewater isolates from all investigated regions) belonging to ST2, a successful widespread *Ab* clone. SNP analyses suggest the relatedness between clinical and hospital wastewater strains (189 − 201 + 202 + 203 + 204, thus less than 20 SNPs), and more intriguing, the relatedness between strains sampled in clinics and urban WWTPs (169 − 184 = 15 SNPs, 187 − 18033O3 = 22 SNPs), suggesting the dissemination of the clinical *Ab* ST2 strains in the wastewaters (Table 5); group III was represented by closely related (26 SNPs) strains isolated from Bucharest hospital and its collecting sewage tank isolates belonging to ST492; group IV included less related (577 SNPs) clinical strains and aquatic strains isolated from Bucharest belonging to ST79; were; group V included less related (> 700 SNPs) wastewater strains isolated from South Romania belonging to ST1; group VI included only environmental strains from the Northern region of the country belonging to ST155 and two related novel STs (Fig. 4).

**Table 5:**
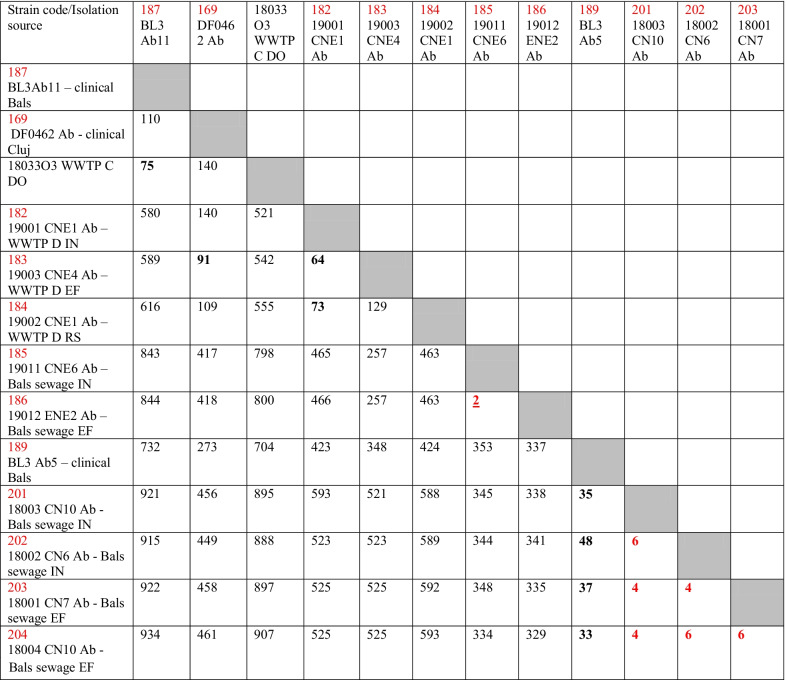
Matrix representation of calculated SNPs distances between the closely related ST2 *A. baumannii* strains

Values in red highlights less than 20 SNPs. Values in bold highlight less than 100 SNPs between the related strains

Pangenome analyses performed on 34 WGS *Ab* strains and 71 selected genomes from the NCBI database, supported also by conjugation assays revealed clear dissemination of the same circulating clones from the hospital units into different aquatic compartments [i.e., ST2 encountered in Bucharest hospital unit and the corresponding sampled WWTP carrying the same CP encoding gene (*bla*_OXA-23_ or *bla*_OXA-72_) in *Ab* strains; ST2 carrying *bla*_OXA-72_ gene in *Ab* strains from Târgoviște hospital unit and *bla*_OXA-23_ in the EF of the corresponding WWTP E] and similarities with other international ST2 clones (Additional file 3: Fig. S1).

**Fig. 5:**
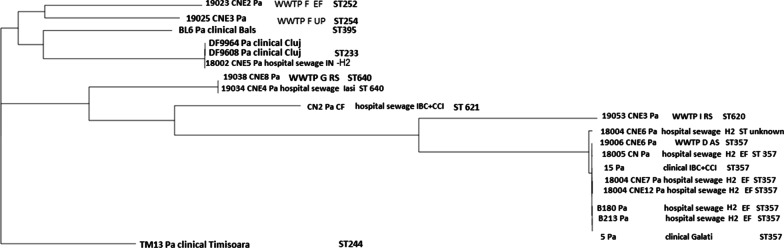
Phylogenetic tree of clinical and wastewater *Pa* strains

The *Pa* strains (Fig. 5) were also grouped in six phylogenetic groups: group I included wastewater isolates from Timișoara, and one clinical strain from the Bucharest hospital that belonged to three singleton STs (ST252, ST254 and ST395); group II comprises clinical strains from Central Romania (Cluj hospital) and one collecting sewage tank from a hospital unit in Bucharest that belonged to the epidemic clone ST233; group III included strains isolated from Iași hospital sewage and its collecting WWTP G belonging to ST640; group IV was represented by one Bucharest hospital unit collecting sewage tank isolate belonging to ST621; group V included wastewater strains from central Romania belonging to ST620; group VI contained the majority of the strains (clinical and wastewater isolates from South and East Romania) belonging to the widespread ST357 and one unknown ST (Fig. 5).

The spread from the hospital unit into the natural aquatic recipient was observed in some cases, i.e.: for an epidemic clone isolated from Galați hospital unit and the receiving WWTP D (ST357 carrying the *bla*_VEB-9_ ESBL encoding gene);two ST640 strains isolated Iași hospital and sludge from the Iași urban WWTP (32 SNPs, thus below the proposed threshold of 37 SNPs). The other *Pa* strains are more diverse, even within the same clone, the strains being more distantly related (> 100 SNPs); this fact was also suggested by the difference between the core genome (4716 genes) and the pan genome (12,395 genes) calculated for all the *Pa* strains included in this study.

## Discussion

Hospitals are a concentrated source of MDR bacteria, which besides having clinical consequences (treatment options are limited and expensive), can be released in wastewater and finally into the environment [45]. Previous studies have revealed the presence of β-lactams, tetracyclines, quinolones and sulfonamides resistance genes in both natural and polluted aquatic environments, indicating that these determinants are released from clinical into aquatic environments, and then further disseminated to opportunistic pathogens [46]. Therefore, rapid identification of high-risk clones is essential for isolating infected patients, preventing the spread of resistance and improving the antimicrobial treatment. This requires the knowledge of the genetic environment and the carrying platforms of ARGs, as well as the development of new methods for assessing the spreading potential of ARGs mediated by mobile genetic elements in both aquatic and hospital environments.

In Romania, in contrast to clinical studies [27, 28, 47–61], there is little information available on the epidemiology of AMR reservoirs in the environment, especially in polluted water and rivers. We have previously shown that MDR, CP and ESBL-producing *K. pneumoniae* isolated from clinics, hospital wastewater and urban WWTPs from different regions of the country exhibit multiple antibiotic and antiseptic resistance, as well as virulence genes, with the ST101 clone being the most frequently encountered in all sampling sites [62]. Also, we have demonstrated the spread of *K. pneumoniae* ST101 from hospital to wastewater influent and its persistence in the wastewater effluent after the chlorine treatment, suggesting its dissemination in the community and in different aquatic compartments [63]. Our previous research showed that enterococci and *Enterobacterales* strains in four Romanian natural aquatic fishery lowland salted lakes from Natura 2000 Network carried a high diversity of resistance markers correlated with class 1 integrons [64]. Other authors described tetracycline and sulfonamides ARGs in the WWTP and the receiver river from northwestern Romania and demonstrated that some ARGs, such as *bla*_VIM_ and *bla*_SHV_ could persist in the chlorinated hospital wastewater, being detected both in the influent and chlorinated effluent [65, 66].

The purpose of this study was to characterize the AMR profiles and clonality of two of the most dangerous ESKAPE pathogens, *Ab* and *Pa*, isolated for two consecutive years from hospital settings, hospital collecting sewage tanks and the receiving WWTPs from three different geographical regions of Romania. The clinical and environmental *Ab* isolates recovered from different geographical regions of Romania revealed high AMR and MDR levels. In another study, from a significant number of groundwater, surface water, and soil samples from Hungary, there were isolated different *Acinetobacter* species (i.e., *A. baumannii*, *A. johnsonii*, *A. gyllenbergii* and *A. beijerinckii*, with 8.10% of *A. beijerinckii*) exhibiting an MDR phenotype [67].

In our study, imipenem-resistant *Ab* and *Pa* strains were also resistant to other classes of clinically important antibiotics, including quinolones (ciprofloxacin) and aminoglycosides (gentamicin). MDR was defined according to Magiorakos et al. [45], as non-susceptibility to at least one agent in three or more antimicrobial classes. The phenotypic resistance profiles and MDR rates have largely varied by sampling point and by geographic location. The highest MDR rates in aquatic isolates were recorded in Galați WWTP (D) that could be explained by the location of this county on the lower course of Danube River. The Danube River is considered the most important non-oceanic body of water in Europe and the “future central axis for the European Union”, with its Danube Delta included in the Biosphere Reserve and Ramsar Sites lists. The Danube River crosses ten countries, so this basin represents an optimal pool for resistant pathogens and anthropogenic pollutants dissemination and accumulation throughout large and distant areas, being assigned as a reservoir of AMR. The following two locations with high MDR rates in the aquatic isolates were Bucharest (H) (the capital and largest city) and Târgoviște (E), both located in the Southern part of the country.

The most frequently CPs encountered in clinical and environmental *Ab* strains were OXA-23 and OXA-24*,* while the ESBLs were represented by SHV, TEM and GES. Hrenovic et al., in 2016 investigated the AMR of *Ab* recovered from the IN and the final EF of a municipal WWTP in Zagreb, Croatia and revealed that 66.66% of the *Ab* isolates were positive for the acquired CP *bla*_OXA-23_ and *bla*_OXA-24_ [68]. Previous data has also indicated the presence of different *A. baumannii complex* species with MDR phenotypes isolated from environmental samples in Hungary [67]. For *Pa* clinical strains, the following CPs and ESBLs have been detected: IMP, VIM, NDM, VEB, GES and TEM.

WGS bioinformatic analysis of *Ab* strains highlighted that the international clone ST2 is broadly spread in our country (Additional file 3: Fig. S1), with 56% of the analyzed *Ab* strains belonging to this clone. Two ST2 strains were included in ST2 branch since the ST492 is a single locus variant of ST2 [61]. The other STs (in the set highlighted with grey in Additional file 3: Fig. S1) have phylogenetic relationships in accordance with the reference sequences, meaning that the whole cluster highlighted with grey is not homogenous, due to random selection of the reference sequence for the phylogenetic analysis. Therefore, the following STs belong to the cluster (in the same order as in the phylogenetic tree): ST499, ST78, unknown, unknown, ST155, ST622, ST46, ST16, ST40, ST403, unknown, ST429, unknown, ST71, ST113, ST25, unknown, ST10, ST10, ST10, ST108, ST514, unknown (Additional file 3: Fig. S1). Worldwide CP producers are mostly associated with international clone II and OXA-23 [61, 69, 70]. Other clinical and wastewater isolates belonged to ST636, ST1, ST79, ST492 and ST2 and were correlated with OXA-72. In Zagreb (Croatia) carbapenem-resistant *Ab* positive for *bla*_OXA-23_ recovered from different sampling points of a WWTP and the sewage of a nursing home belonged to the international clonal lineage IC2, the OXA-72 producers belonged to IC1, while the susceptible ones were unclustered [71, 72]. The OXA-23, OXA58, and OXA-72 CPs linked to ST2 in hospital environments have also been reported in other countries such as Croatia, Serbia, Bosnia and Herzegovina [73–76].

The WGS analysis of *Pa* strains has shown that ST357 was correlated with IMP-13 in one clinical strain; in the sewage effluent, ST357 was correlated with VEB-9 and with both IMP-13 and VEB-9; the epidemic clone ST233 was correlated with VIM-2 in clinical and wastewater *Pa* strains; ST640 with TEM-40 in hospital sewage and WWTP; ST621 with IMP-13 in hospital sewage; ST620 with GES-1 in WWTP, while an unknown ST was correlated with VIM-2 in a sewage strain. The singletons ST252, 254, 244, 395 were not CP or ESBL producers.

A class 1 integron (qacE∆1 integron-associated gene) was present in most of the identified *Ab* clones. This association could be the result of co-selection processes due to the spread of successful clones (such as ST2, ST636 and ST1 *Ab*) that were selected by antibiotic treatment in the hospital settings and were able to accumulate various CPs and ESBLs (i.e., OXA-23, OXA-72, TEM-12, ADC-30, ADC-74, ADC-73, ADC-81). Class 1 integrons were revealed in 80% of *Pa* strains and 50% of *Pa* belonging to the epidemic clones ST233, ST357 and the ST244.

Since the prevalent clones have a great potential for transmission among patients, the observation that those prevalent clones are correlated with the presence of class 1 integrons suggests that those isolates could be a significant reservoir of ARGs and can persist in the environment.

One of the limitations of this study arises from the fact that we have selected, using antibiotic supplemented culture media, only the resistant *Ab* and *Pa* strains, while the total population structure of these pathogens (including the antibiotic-sensitive strains) could not be assessed by this approach. However, we have isolated non-MDR strains in few cases (e.g., *Ab* strains from Cluj WWTP I and *Pa* strains from Târgoviște, Vâlcea, Iași hospitals and from Iași WWTP G and Timisoara WWTP F), but these isolated were not investigated at the genetic level (Additional file 1: Tables 1 and 2).

## Conclusion

Our study emphasized the presence of carbapenem-resistant MDR *Ab* and *Pa* belonging to international high-risk clones in all investigated sampling points (hospital units, their collecting sewage tanks and the sampled WWTPs) and the clonal dissemination of clinical *Ab* ST2 strains in the wastewaters. The reported data highlight the importance of the screening for acquired AMR in the environment and could provide important knowledge for monitoring the ARB and ARGs transmission from hospital into water bodies.

## Supplementary Information

**Additional file 1**. Tables S1 and S2 AR profiles in clinical and wastewater *Ab* and* Pa* strains.

**Additional file 2**. Table S3 ARGs in clinical and wastewater *Ab* strains isolated in 2019.

**Additional file 3**. Fig. S1 Phylogenetic tree of 105 genomes based on pangenome analysis. It was observed that 5 ST2 resistant clinical strains (GCF_000186645.1, GCF_000302075.1, GCF_018928195.1, GCF_000189655.1 and GCF_005819175.1) of the 71 reference strains were closely related with sequences belonging to strains isolated from intra-hospital infections in H2, Târgoviste and Cluj and from wastewater strains isolated from the collecting sewage tanks of hospital H2 Târgoviste and WWTP D and G.

## Data Availability

All data analyzed or generated during this study are included in this published article and its Supplementary Information files. Any additional information is available from the corresponding author on reasonable request.

## Abbreviations

AMR: Antimicrobial resistance
WWTPs: Wastewater treatment plants
ARB: Antibiotic resistant bacteria
ARGs: Antibiotic resistance genes
MDR: Multi-drug resistant
CP: Carbapenemase
ESBL: Extended spectrum β-lactamase
*Ab*: *Acinetobacter baumannii*
*Pa*: *Pseudomonas aeruginosa*
WGS: Whole-genome sequencing
MGEs: Mobile genetic elements
HGT: Horizontal gene transfer
JPIAMR: Joint programme initiative on antimicrobial resistance
CDC: Centers for disease control and prevention
CHDLs: Carbapenem-hydrolyzing class D β-lactamases
MBL: Metallo-β-lactamases
VIM: Verona integron-encoded MBL
IMPs: Imipenemases
NDM: New Delhi metallo-β-lactamase
AIM: Australian imipenemase
CAM: Central alberta MBL
DIM: Dutch imipenemase
FIM: Florence imipenemase
GIM: German imipenemase
HMB: Hamburg MBL
SPM: São Paulo MBL
SIM: Seoul imipenemase
VEB: Vietnamese extended-spectrum β-lactamase
PER: *Pseudomonas* extended-resistant
TEM: Temoneira patient’s name
SHV: Sulfhydryl variable enzyme
CTX-M: Cefotaxime first isolated in munich carbapenemase
GES: Guyana Extended Spectrum β-lactamase
WWTP C and H: Bucharest WWTP
WWTP E: WWTP Târgoviște
WWTPJ: WWTP Râmnicu-Vâlcea
WWTP I: WWTP Cluj
WWTP F: WWTP Timișoara
WWTP G: WWTP Iași
WWTP D: WWTP Galați
EF: Effluent
IN: Influent
AS: Activated sludge
RS: Returned sludge
CLSI: Clinical and laboratory standards institute
RIF: Rifampicin
MLST: MultiLocus sequence typing
MEM: Meropenem
IMP: Imipenem
CAZ: Ceftazidime
ATM: Aztreonam
FEP: Cefepime
TZP: Piperacillin-tazobactam
CIP: Ciprofloxacin
GEN: Gentamicin
AK: Amikacin
MH: Minocycline
SAM: Ampicillin sulbactam
H: Hospital
SNP: Single nucleotide polymorphism
